# The preventive effect of probiotic *Lactobacillus plantarum* X86 isolated from raw milk on *Staphylococcus aureus*-induced mastitis in rats

**DOI:** 10.3389/fvets.2025.1476232

**Published:** 2025-03-10

**Authors:** Xiulan Xie, Mei Cao, Shiying Yan, Haihui Gao, Yuwei Yang, Jiayi Zeng, Gang Zhang, Jian Zhao

**Affiliations:** ^1^Institute of Animal Science, Ningxia Academy of Agriculture and Forestry Sciences, Yinchuan, China; ^2^Key Laboratory of Biological Resource and Ecological Environment of Chinese Education Ministry, College of Life Sciences, Sichuan University, Chengdu, China; ^3^Core Laboratory, School of Medicine, Sichuan Provincial People's Hospital Affiliated to University of Electronic Science and Technology of China, Chengdu, China; ^4^Key Laboratory of Ministry of Education for Protection and Utilization of Special Biological Resources in Western China, Department of Biochemistry and Molecular Biology, College of Life Science, Ningxia University, Yinchuan, China

**Keywords:** raw milk, *Lactobacillus plantarum* X86, *Staphylococcus aureus*, rat mastitis model, anti-inflammatory

## Abstract

Mastitis is the most common and challenging disease that affects dairy animal welfare and causes huge economic loss in dairy industry globally. Conventional antibiotic treatment of mastitis raised the drug resistance and unsuccessful therapy. As an alternative approach, probiotic lactobacilli had shown multifunctional effects against diseases. Lactobacillus strains against mastitis are worth screening and evaluating. In this study, milk-derived Lactobacillus spp. from Ningxia, China were screened *in vitro* and the anti-mastitis effect of a candidate strain was evaluated through a *Staphylococcus aureus*-induced rat mastitis model. The results showed that *Lactobacillus plantarum* X86 exhibited a high adhesion rate of MAC-T cells, presented the best probiotic properties, and demonstrated anti-*S. aureus* effects *in vitro* through comprehensive assessment. Furthermore, *L. plantarum* X86 alleviated pathological damage to the mammary gland, liver, and colon, inhibited the mRNA expression of pro-inflammatory cytokines factors IL-1β, IL-6, and TNF-*α* in mammary gland tissue; and increased the content of intestine SCFAs in a rat mastitis model induced by *S. aureus*. In conclusion, our results suggested that *L. plantarum* X86 could be a promising probiotic for the prevention and treatment of *S. aureus*-induced mastitis.

## 1 Introduction

Cow mastitis is one of the major diseases in the dairy industry, causing substantial economic losses, reducing milk production and quality, as well as the life span of cows, and increasing the cost of subsequent treatment (1). Currently, a novel perspective suggests that there is a close relationship between the intestinal barrier and mastitis. For example, a study found that subacute ruminal acidosis (SARA) in dairy cows activate systemic inflammatory response and increase the permeability of blood-milk barrier, intestinal barrier and rumen barrier, which can cause mastitis (2). Mice with dysregulated intestinal flora exhibited an increase in the abundance of enterobacterium and a decrease in the number of SCFAs-producing bacteria, along with enhanced blood-milk barrier permeability and more severe *Staphylococcus aureus*-induced mastitis. This situation can be reversed through fecal microbiota transplantation (FMT) in mice with impaired intestinal flora (3). Therefore, enhancing the integrity of the intestinal barrier, and preventing the passage of harmful substances and bacteria through the barrier can influence the health of the mammary gland.

Antibiotics are utilized in dairy cows for the treatment of clinical mastitis and during the dry period, which leads to issues such as bacterial resistance, antibiotic residues and transmission of resistance genes (4). Given the current emphasis within the global livestock and poultry industry on developing “non-resistance products,” there is an urgent need to develop anti-resistance products for the prevention and management of cow mastitis.

Lactic acid bacteria are a group of bacteria that produce a large amount of lactic acid by fermenting carbohydrates. Some lactic acid bacteria are recognized as food safety-grade strains (5). Lactic acid bacteria isolates had exhibited anti-mastitis effects both *in vitro* and *in vivo*, such as inhibiting the adhesion and internalization of mastitis pathogenic bacteria to BMECs (6), inhibiting the growth of pathogenic bacteria (7), and reducing the pathological damage of cells (8–10) or animal models of mastitis through immunomodulatory effects (9) and enhancing the blood-milk barrier (11). Some potential Lactobacillus strains (*L. salivarius* CECT5713 & *L. gasseri* CECT5714) (12), *L. fermentum* CECT5716 (13) and *L. salivarius* PS2 (14) that replaced *Staphylococcus* spp. causing human mastitis were effective in clinical trials. Several studies have showed that Lactobacillus can reduce the abundance of *enterococcus* and *streptococcus* in the milk of dairy cows with mastitis and alleviate the inflammation of dairy cows suffering from mastitis (15, 16). Breast milk and cow milk are important sources of lactic acid bacteria (17, 18). Lactobacilli isolated from breast milk meet some of the main criteria for generally recommended human probiotics, including human origin, a safety history, long-term infant intake, and adaptation to a dairy matrix (19). Hence, it is promising to explore the probiotic lactic acid bacteria strains from milk for the prevention and treatment of mastitis in dairy cows.

Chandler was the first to establish a mouse model of bacterial mastitis (20), which is still utilized today (21, 22). Compared with mice, the milk duct opening of rats is more distinct and has higher operability during modeling, thus it is also widely employed (23, 24). Due to the high cost and non-standardized operation of direct experiments on cows, a rat model of *S. aureus* mastitis was established in this paper to assess the preventive effect and related mechanism of *Lactobacillus plantarum* X86 on mastitis.

Therefore, we hypothesized that the screened milk-derived Lactobacillus strains had an anti-mastitis effect in this paper, which was achieved by enhancing the mammary gland and intestinal barrier. This will provide a basis for alternatives for the prevention and control of bovine mastitis.

## 2 Materials and methods

### 2.1 Ethics statement

Animal experiments were conducted in accordance with the guidelines and regulations by the Institutional Animal Care and Use Committee (IACUC) to ensure the ethical treatment of animals. All procedures involving handing and restraint, gavage, blood collection, and anesthetized were carried out with rigorous ethical standards in place to minimize any potential harm or distress to the rats. The animal experiments had been approved by the Ethics Committee of the College of Life Sciences, Sichuan University (SCU2203016).

### 2.2 Animal grouping and experimental design

Twenty-four pregnant SD rats were individually housed in a barrier system at 22°C and 40–70% humidity (Chengdu Lilai biotechnology Co., Ltd.). After a 7-day adaptation period (14 ± 2 days after pregnancy), the rats were randomly assigned to three groups: Control (*n* = 8), X86 + *S. aureus* (*n* = 8) and *S. aureus* (*n* = 8). Rats in X86 + *S. aureus* group received daily gavage of 0.5 mL of *L. plantarum* X86 at a concentration of 1 × 10^9^ CFU/mL, while rats in control and *S. aureus* groups received an equivalent volume of normal saline until the 14th day post-delivery. Subsequently, on the following day after completion of gavage, mastitis models were established in both the X86 + *S. aureus* and *S. aureus* groups.

### 2.3 Experimental strains and cells

*Lactobacillus rhamnosus* LGG was obtained from commercially available probiotic products. *Lactobacilli* spp. was isolated from raw milk collected from commercial farms in Ningxia Hui Autonomous Region, China. The commercial dairy farms are located in the north (Farm A, N106.69, E39.12), mid (Farm B, N106.04, E38.42 and Farm C, N106.07, E38.67), and south (Farm D, N106.29, E37.75; Farm E, N106.30, E37.58; and Farm F, N106.24, E37.59) regions of Ningxia Province in China. The raw milk samples were collected from 2018 to 2019. The mammary tissue of the cows did not have visible signs of clinical mastitis, such as swelling or redness. Milk samples were collected from one teat for each cow. The sampling methods adhered to standard recommendations. In brief, the first streams of milk were discarded, and the teats were subsequently exposed to iodine tincture for 30 s and dried with individual towels by farm veterinarians. Subsequently, the first streams of milk were discarded and the milk samples were collected. Approximately 30 mL of milk was collected into a 50 mL sterile centrifuge tube and stored at −20°C. *S. aureus* strains SA2 and SA6 were isolated from the raw milk of cows with mastitis in the Ningxia region, while *S. aureus* ATCC29213 was preserved in the laboratory; MAC-T cells were gifted by Associate Professor Gao Jian, College of Veterinary Medicine, China Agricultural University.

### 2.4 Isolation and identification of lactic acid bacteria from raw milk

Raw milk samples were collected from large-scale ranches in Ningxia and used as resources. The milk samples were then inoculated into MRS liquid medium (Qingdao Hope Bio-Technology Co., Ltd., China) at a 3% inoculum volume ratio in a sterile tube and cultured for 48 h at 37°C. Afterwards, the culture medium was diluted by 10-fold, and the bacterial solution was spread on MRS agar plates, which were then incubated at 37°C for 48 h. Subsequently, several colonies were randomly selected and subjected to two consecutive rounds of inoculation into MRS agar medium (Qingdao Hope Bio-Technology Co., Ltd., China) for purification.

The isolates were identified through 16S rRNA sequencing, following a process in which a single colony was selected and immersed in 100 μL of 20 mM NaOH solution, then boiled in hot water for 10 min. The solution was centrifuged at 12000 rpm for 2 min, and the supernatant used as template for PCR amplification. Universal bacterial 16S rRNA primers (27F: AGAGTTTGATCCTGGCTCAG, 1492R: CTACGGCTACCTTGTTACGA) were utilized for PCR amplification, consisting of 20 μL of 2 × Taq PCR PreMix (Vazyme Biotech Co., Ltd., Nanjing, China) along with 1 μL each of forward and reverse primer (10 μM), 2 μL template, and finally adding ddH_2_O up to a total volume of 40 μL. The PCR protocol included an initial denaturation at 94°C for 5 min, followed by 35 cycling at 94°C for 30 s, 56°C for 30 s, and 72°C for 30 s, and with a final extension step at 72°C for 2 min. The PCR products were sent to Shanghai Sangon for sequencing. Sequence alignment was performed using the NCBI^1^, and those with homology of 97% or higher were considered to the same species.

The isolates were cultured in MRS liquid medium at pH 3.0 and incubated at 37°C and 180 rpm for 18–24 h for initial screening. Strains exhibiting obvious turbidity in the bacterial solution were chosen for subsequent experiments. Gram staining and microscopic morphological analysis of the bacteria were performed on these isolates. The phylogenetic tree of isolates and the control strain *L. rhamnosus* LGG was conducted using MEGA11 software (25) through the neighbor-joining method.

### 2.5 Microbiological and probiotic property analysis

#### 2.5.1 The growth and pH curve determination

The bacterial growth curve was determined with appropriate modifications based on references (26, 27) as follows: isolates were inoculated in MRS broth at a ratio of 2% and cultured at 37°C, 180 rpm for 48 h. Subsequently, 200 μL of the bacterial solution was added to a 96-well plate every 3 h, with MRS broth used as a control, and the absorbance measured at OD600 nm using a microplate absorbance spectrophotometer (xMarkTM, Bio-Rad, Hercules, California, United States). Furthermore, the pH value of the bacterial solution was measured every 3 h using FE20-FiveEasy pH meter (Mettler Toledo Inc., United States) for drawing the pH curve.

#### 2.5.2 The survival rates of isolates through artificial gastroenteric fluid

The preparation method for artificial gastric and intestine fluid was performed in accordance with the protocol outlined by Huang and Adams (28). The isolates were inoculated into MRS broth at a ratio of 2%, incubated at 37°C and 180 rpm, then centrifuged at 6000 rpm for 10 min. The pellet was washed twice with 5 mL of PBS buffer. Subsequently, the cells were adjusted to a concentration of 1 × 10^9^ CFU/mL in artificial gastric fluid (pH 3.0) and cultured at 37°C and 180 rpm for 3 h. Following this, bacterial fluid was added to artificial intestinal fluid (pH 8.0) in a ratio of 1:9 and cultured at 37°C, 180 rpm for 8 h. Finally, a 10-fold dilution of the bacterial solution was prepared in artificial gastric and intestinal juice, and 1 mL of different concentrations of the bacterial solution was poured onto MRS agar at approximately 40°C. The cells were thoroughly mixed and cultured at 37°C for 48 h, followed by enumeration of colonies on plates. The survival rates of bacteria in artificial gastric fluid and intestinal fluid were calculated using the following formula (Equation 1) provided by Bao et al. (29):


(1) $$\mathrm{Survival}\;\mathrm{rate}\;\left(\%\right)=\mathrm{LogN}1/\mathrm{logN}0\times 100\%$$

where *N*1 = the viable count of lactic acid bacteria cultured in the artificial gastric/intestinal fluid, and *N*0 = the viable count of lactic acid bacteria prior to inoculation.

#### 2.5.3 The survival rates of isolates through bile salt

The viability of isolates in the culture medium containing bile salt was measured *in vitro* to evaluate their tolerance to bile salt. A volume of 1 mL of isolates (10^9^ CFU/mL) was inoculated into 9 mL of MRS-THIO solution containing 0.3% bovine bile salt and incubated at 180 rpm for 24 h. Agar plate count and survival rate were determined following the method mentioned above.

#### 2.5.4 The *Staphylococcus aureus* growth inhibition assay

##### 2.5.4.1 Preparation of the CFS of isolates

The preparation of cell free supernatant (CFS) was conducted as follows: the MRS Broth medium was filled to 75% capacity (37.5 mL/50 mL), and the activated strain medium was inoculated at a ratio of 2% and incubated at 37°C for 48 h. The culture solution was then centrifuged at 6000 rpm for 10 min, and the supernatant was transferred into a sterile centrifuge tube and stored at 4°C until further use.

##### 2.5.4.2 The growth inhibition assay of CFS against *Staphylococcus aureus*

The experiment was conducted in accordance with the methods described by Bai et al. (30). CFS was diluted twofold with LB liquid medium (Qingdao Hope Bio-Technology Co., Ltd., China) to achieve concentrations of 1, 0.5, 0.25 and 0.125. 100 μL. Subsequently, 100 μL of *S. aureus* (2 × 10^5^ CFU/mL) suspension and of 100 μL of CFS at different concentrations were added to a 96-well plate at 37°C for 24 h. The absorbance was measured at OD600 nm using a microplate absorbance spectrophotometer (xMark™, Bio-Rad, Hercules, California, United States). LB liquid medium served as the negative control while *S. aureus* suspension served as the positive control. The inhibition rate was calculated using the following formula (Equation 2):


(2) $$\begin{array}{l} \mathrm{Inhibition rate}\;\left(\%\right)\\ =\frac{\left[\left(A_{OD600}-C_{OD600}\right)-\left(B_{OD600}-C_{{OD}_{600}}\right)\right]}{A_{OD600}-C_{OD600}}\times 100\% \end{array}$$

where A_OD600_ = the mean value of positive control wells, B_OD600_ = the mean value of sample wells, and C_OD600_ = the mean value of negative control wells.

##### 2.5.4.3 Inhibition assay of CFS against biofilms of *Staphylococcus aureus*

The inhibition of *S. aureus* biofilm by the CFS of isolates was carried out in accordance with the protocol described by Bai et al. (30). The CFSs of isolates were twofold serially diluted with LB liquid medium containing 1% glucose to achieve concentrations of 1, 0.5, 0.25 and 0.125. Subsequently, 100 μL *S. aureus* (2 × 10^6^ CFU/mL) was added to a 96-well plate, followed by the addition of 100 μL of each concentration of CFS and incubated at 37°C for 24 h to allow bacterial biofilms formation. After discarding the bacterial solution, the wells were washed twice with 200 μL PBS buffer to remove plankton cells. Then, a solution containing 200 μL of 0.4% crystal violet was added for 5 min and subsequently washed twice with distilled water (200 μL). Finally, a solution containing 200 μL of glacial acetic acid (20%) was added and incubated at 25°C for 30 min to dissolve the biofilm. LB liquid medium containing 1% glucose served as a negative control while *S. aureus* suspension (1 × 10^6^ CFU/mL) served as a positive control for measuring absorbance at OD570 nm using a microplate absorbance spectrophotometer (xMark™, Bio-Rad, Hercules, California, United States). The inhibition rate was calculated using the following formula (Equation 3):


(3) $$\begin{array}{l} \mathrm{Inhibition rate}\;\left(\%\right)\\ =\frac{\left[\left(A_{OD570}-C_{OD570}\right)-\left(B_{OD570}-C_{{OD}_{570}}\right)\right]}{A_{OD570}-C_{OD570}}\times 100\% \end{array}$$

where A_OD570_ = the mean value of positive control wells, B_OD570_ = the mean value of sample wells, and C_OD570_ = the mean value of negative control wells.

#### 2.5.5 Antibiotic resistance test

The broth microdilution method was employed to determine the drug resistance phenotype of lactic acid bacteria (26). The antibiotic stock solution was diluted using a twofold dilution method to obtain 12 concentrations commonly used in the determination antibiotic resistance phenotypes. Subsequently, 100 μL serial concentrations of the antibiotic solution were added to 96-well plates, followed by the addition of 100 μL suspension of lactic acid bacteria to be tested (2 × 10^5^ CFU/mL). The cells were then cultured at 37°C for 24 h with shaking at medium speed for 1 min and measurement of OD600. A negative control using 200 μL MRS broth and a positive control using a lactic acid bacteria suspension (1 × 10^5^ CFU/mL) were included. The cut-off value of antibiotic resistance of lactic acid bacteria as defined by the European Food Safety Authority (EFSA) literature (31) was adopted, along with additional references (32–34). The cut-off values of antibiotic resistance by the broth microdilution method for various genera of lactobacilli were shown in Supplementary Table S1. Results were judged according to the following criteria: sensitivity (S): ≤ cut-off value; drug resistance (R): > cut-off value.

#### 2.5.6 Adhesion of isolates to MAC-T cells

The ability of the adherence into host cells have a key role to provide the foundation for Lactobacillus to exert its benefits. The bovine mammary epithelial cell line (MAC-T) has been widely used for adhesion and invasion assays (6, 35). Therefore, the adhesion capability to MAC-T was assessed of *L. plantarum* X86 in this study. The adhesion of lactic acid bacteria to MAC-T cells was assessed following the protocol described in Bouchard et al. (6): MAC-T cells were seeded at a density of 2 × 10^5^ cells/well in 12-well plates and incubated at 37°C in a 5% CO_2_ for 24 h. Subsequently, the cells were washed twice with PBS and exposed to lactic acid bacteria at a multiplicity of infection (MOI) of 2000:1, with a concentration of 5 × 10^8^ CFU/mL, for adhesion. The co-culture was maintained at 37°C with 5% CO_2_ for 1 h. Following this incubation period, nonadherent lactic acid bacteria were removed by washing the MAC-T cells four times with PBS. Cell digestion was then carried out by adding 0.25 mL of trypsin (0.05%) to each well and incubating at 37°C for 10 min. Lysis of the cells was achieved by adding 100 μL of Triton (0.01%). Finally, all liquid from the wells was collected and cell counting performed using the plate counting method as previously described.

#### 2.5.7 Evaluation of the biological characteristics of lactic acid bacteria

Based on the findings from the aforementioned experiments, the biological characteristics of lactic acid bacteria were categorized into five indicators: (1) growth (including growth curve and acid production curve); (2) fundamental probiotic benefits (encompassing artificial intestinal, gastric juice, and bile salt tolerance); (3) inhibitory effects on *S. aureus* (comprising growth and biofilm inhibition of *S. aureus* strains); (4) adhesion to MAC-T cells; and (5) resistance of isolates to antibiotics. Each of these five indicators is allocated 20 points, resulting in a total score of 100 points. Please refer to Supplementary Table S2 for detailed scoring criteria.

### 2.6 The anti-mastitis assay of Lactobacillus strain in rats

#### 2.6.1 Establishment of the *Staphylococcus aureus*-induced mastitis model in rats

The rat mastitis model was established following the protocol outlined by Chandler (20). The specific procedures were as follows: (1) offspring rats were removed 2 h prior; (2) rats were anesthetized with isoflurane gas and maintained under anesthesia; (3) nipple and surrounding skin were disinfected with a 75% ethanol cotton ball, after identifying the opening of the breast duct under the operating microscope, the nipple was gently clamped by forceps, and a 30G syringe needle was inserted. Subsequently, the nipple was gently lifted by forceps and covered by the needle to inject liquid into the breast duct. After removing the needle, there should be no liquid exudation from the breast catheter and no obvious swelling upon touch; (4) The fourth and fifth pairs of mammary glands were perfused with 100 μL *S. aureus* (10^6^ CFU/mL), while the Control group received an equivalent volume of normal saline; (5) Rats were returned to their cages for natural recovery and continued feeding; and (6) After 24 h, rats were anesthetized with intraperitoneal pentobarbital sodium for dissection and specimen collection.

The blood from the abdominal aorta was collected and the serum was separated, then stored at −20°C. The tissues were immediately preserved in liquid nitrogen and subsequently transferred to −86°C for future use. Breast tissues, jejunum and colon tissues were aseptically obtained for pathological examination. The specimens were promptly fixed in fixative solution with an exchange with 2 h. Tissue specimens were fixed in neutral formaldehyde, followed by routine paraffin sections stained with hematoxylin eosin staining.

#### 2.6.2 ELISA for the serum secretion of cytokines and MPO

Cytokines play a significant role in the process of breast inflammation, and exist in the form of a network, mutually influencing and inducing each other. Pro-inflammatory cytokines, such as TNF-*α*, IL-1β, and IL-6 play crucial roles in regulating infection and inflammation. Myeloperoxidase (MPO) activity, a marker of neutrophils, serves as an indicator of inflammation in mammary tissue. To a certain degree, the content of TNF-α, IL-1β, and IL-6, as well as the activity of MPO are regarded as indicators of inflammation in mammary tissue (36, 37). The concentrations of pro-inflammatory factors (IL-1β, IL-2, IL-6 and TNF-α) and MPO in serum were determined using the ELISA method according to the instructions of ELISA kit (Chengdu Pengshida company) and MPO kit (Nanjing Jiancheng Co., Ltd., China).

#### 2.6.3 The mRNA expression of cytokines in rat tissues

The gut tissues (colon and jejunum), mammary gland, and liver tissues were aseptically collected from rats for the assay of inflammatory cytokines mRNA expression. The tissues were homogenized and total RNA was extracted using the RNAprep pure Tissue Kit (JIANSHI Biotechnology Co., Ltd., Beijing, China) according to the protocol. The quantification of RNA was measured using the Ultralow volume spectrometer BioDrop uLite+ (Biochrom Ltd., Cambridge, United Kingdom). Subsequently, cDNA was synthesized using PrimeScript™ RT reagent Kit (TaKaRa Biotechnology Co., Ltd., Dalian, China) with 0.5 μg of total RNA input normalized. qRT-PCR primers were designed using NCBI Primer Blast and an online primer design tool.^2^ The primer list was provided in Table 1. The qRT-PCR reaction system included Hieff UNICON^®^ Universal Blue qPCR Green Master Mix (2×) (Yeasen Biotechnology Co., Ltd., Shanghai, China) 10 μL, Primers (10 μM) 0.4 μL each, template (10-fold dilutions of cDNA) 3.0 μL, ddH_2_O was added to make up to 20 μL in total volume. The qRT-PCR procedure consisted of pre-denaturation at 95°C for 2 min for 1 cycle, followed by 40 cycles of denaturation at 95°C for 10 s and annealing at 60°C for 30 s; the melting curve followed the instrument default settings. Data analysis was performed using QuantStudio™ Design & Analysis Software and changes in expression were calculated using the 2^−ΔΔCt^ method with three repeated experiments conducted.

**Table 1:** Sequences of the primers used for qRT-PCR.

Genes	Primer sequence	Amplicon length(bp)	Ref Seq
GAPDH	Forward: TCTCTGCTCCTCCCTGTTCT	107	NM_017008.4
	Reverse: CGATACGGCCAAATCCGTTC		
IL-1β	Forward: AGCTTTCGACAGTGAGGAGA	138	NM_031512.2
	Reverse: TGTCGAGATGCTGCTGTGAG		
TNF-α	Forward: ATGGGCTCCCTCTCATCAGT	106	NM_012675.3
	Reverse: GCTTGGTGGTTTGCTACGAC		
IL-6	Forward: ACTTCCAGCCAGTTGCCTTCTTG	110	NM_012589.2
	Reverse: TGGTCTGTTGTGGGTGGTATCCTC		
ZO-1	Forward: GCCAGCTTTAAGCCTCCAGA	144	NM_001106266.1
	Reverse: TGGCTTCGCTTGAGGTTTCT		
Occludin	Forward: GGGGCGCAGCAGGTCT	181	NM_031329.3
	Reverse: GTGCATCTCTCCGCCATACA		
Claudin-1	Forward: TGCCCTACTTTCCTGCTCCTGTC	115	NM_031699.3
	Reverse: CTTCCTTCGCCGCTGTCACAC		

#### 2.6.4 SCFAs content during the *Staphylococcus aureus*-induced mastitis model

SCFAs, primarily composed of acetic acid, butyric acid and propionic acid, play a pivotal role in immune regulation and inflammatory states (38, 39). A negative correlation was identified between the level of short-chain fatty acids (SCFAs) in the gut and the severity of mastitis (3). To evaluate the potential anti-mastitis mechanism of *L. plantarum* X86, the content of SCFAs during the *S. aureus* mastitis model was measured. The detection of gut SCFAs in rats was conducted using GC–MS. Fecal samples were aseptically collected and 25 mg fecal sample were added to 500 μL water (containing 0.5% phosphoric acid), followed by freezing and grinding twice for 3 min (50HZ), sonicated for 10 min, and centrifugation at 13000 g for 15 min at 4°C. Subsequently, the supernatant was mixed with 0.2 mL n-butyl alcohol solvent (containing internal standard 2-ethylbutyric acid at a concentration of 10 μg/mL) and vortexed for 10s, followed by sonication at 4°C for another 10 min and centrifugation at 13, 000 g at 4°C for 5 min. Finally, the supernatant transferred into the injection vial for analysis using the 8,890-7000D Triple Quad GC/MS (Agilent Technologies Inc., CA, United States). The Masshunter quantitative software was utilized for automatic identification and scoring with manual examination assistance. The concentration of each sample was calculated using a standard curve, and the actual content of SCFAS in the sample was determined accordingly.

### 2.7 Statistical analysis of data

GraphPad8.0 software was utilized for statistical analysis and graph drawing. The two-tailed *t* test was employed to calculate the *p* value for intergroup differences, while one-way ANOVA was used for multiple comparisons. All *p* values were calculated at the 95% confidence level; where *p* > 0.05 was considered no significant, *p* < 0.05 as significant, and *p* < 0.01 as extremely significant.

## 3 Results

### 3.1 Bacterial isolation, identification, and preliminary screening

**Figure 1:**
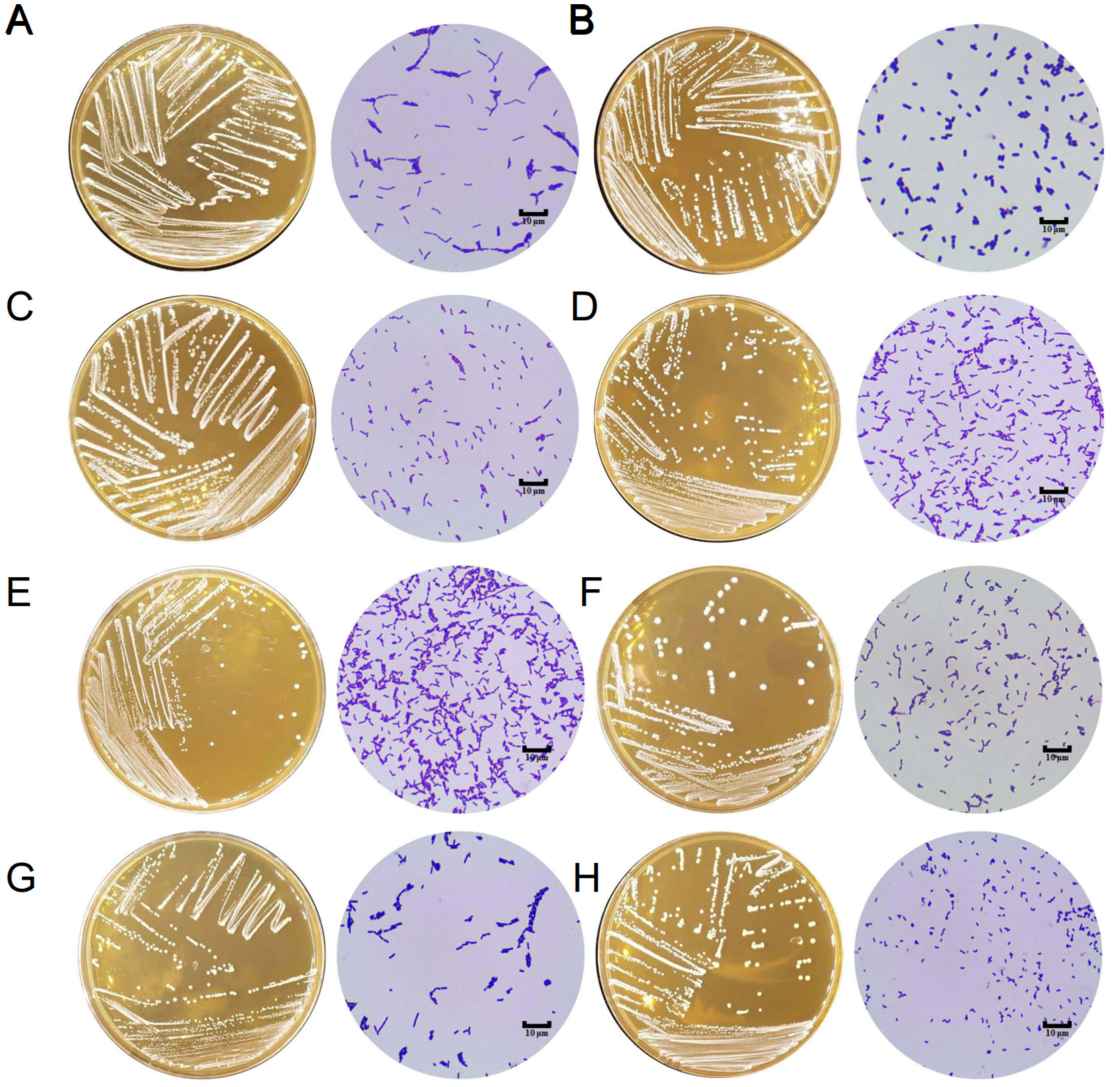
The morphology of Lactobacillus isolates on MRS agar and under microscope (1000×). **(A)** *L. rhamnosus* LGG. **(B)** *L. plantarum* X86. **(C)** *L. rhamnosus* X130. **(D)** *L. paracasei* X133. **(E)** *L. paracasei* X135. **(F)** *L. paracasei* X145. **(G)** *L. plantarum* X275. **(H)** *L. plantarum* X277.

**Figure 2:**
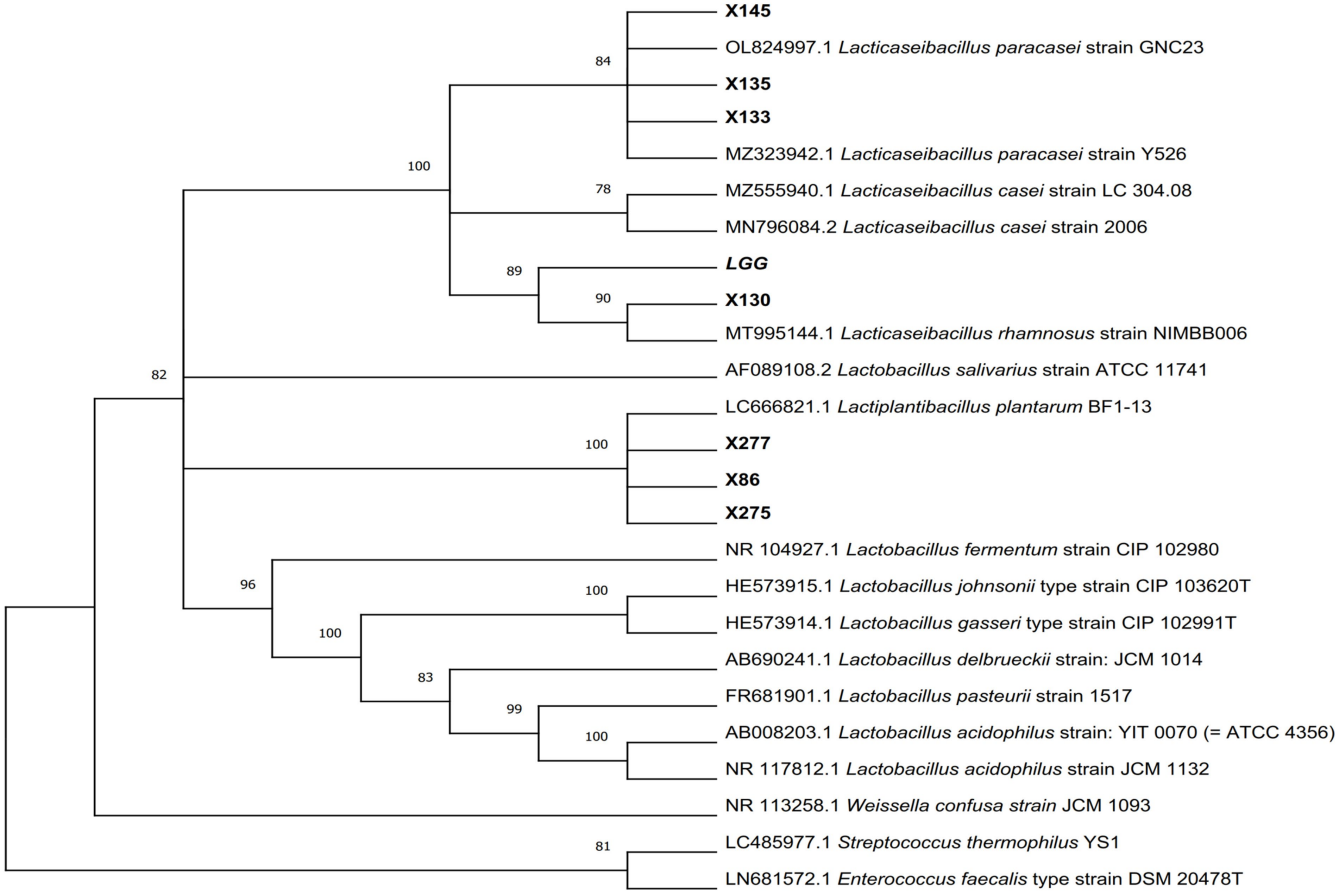
The phylogenetic tree of Lactobacillus isolates was constructed using Neighbor-Joining methods in MEGA11 software, with p-distance used for evolutionary distance based on 16S rRNA gene sequence. The number adjacent to each branch node represents the Bootstrap value, where a bootstrap value >70 indicates a reliable branch. A longer evolutionary branch length signifies more changes in the corresponding species or genes.

**Figure 3:**
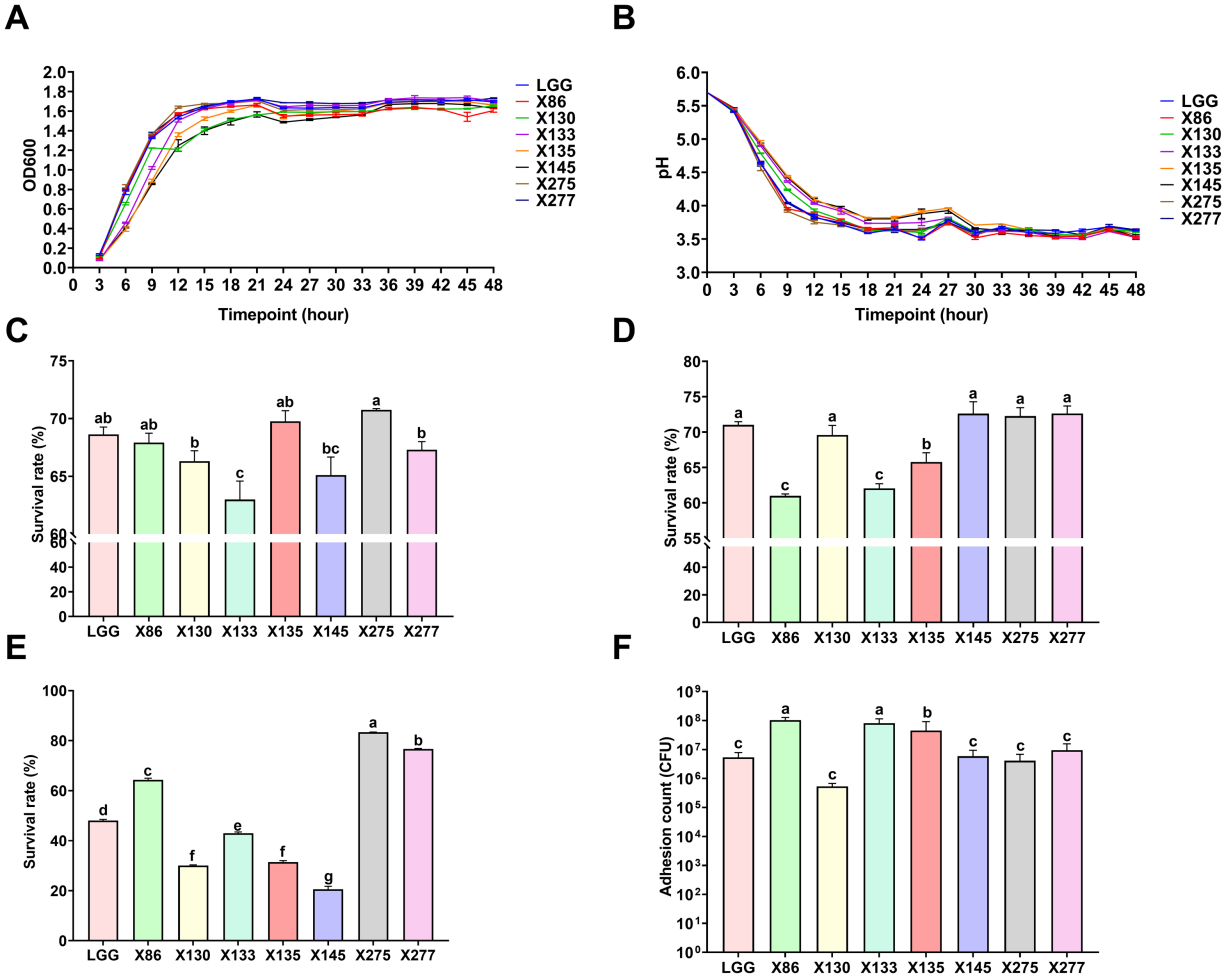
The basic characteristics of Lactobacillus ssp. **(A)** The growth curve of Lactobacillus ssp. in MRS Liquid medium. **(B)** The pH curve of Lactobacillus ssp. in MRS liquid medium. **(C)** The survival rate of Lactobacillus ssp. through the artificial gastric juice. **(D)** The survival rate of Lactobacillus ssp. through the artificial intestinal fluid. **(E)** The survival rate of Lactobacillus ssp. through the bovine bile salt. **(F)** Adhesion count of Lactobacillus spp. to MAC-T cells. The data were presented as Mean ± SD (*n* = 3), and Anova was employed to assess inter-group differences. Significant differences were indicated by distinct shoulder marks (*p* < 0.05), while the absence of significance difference was denoted by identical shoulder markers.

A total of 214 lactic acid bacteria strains were isolated and identified from 100 raw milk and cow colostrum samples (Supplementary Table S3). Among those, 12 strains successfully passed the initial screening in artificial gastrointestinal juice and bile salt, exhibiting vigorous grown *in vitro*. Subsequently, seven strains were selected (Table 2; Figure 1) based on their repeated occurrence. As shown in Figure 2, X86, X275 and X277 clustered into one branch with the closest evolutionary distance to *L. plantarum* BF1-13 on the phylogenetic tree, thus being identified as *L. plantarum*. Similarly, X130 was closely related to *L. rhamnosus* NIMBB006 and was therefore identified as *L. rhamnosus*; while X133, X135 and X145 clustered with *L. paracasei* strain Y526 were identified as *L. paracasei*. *L. plantarum* X86, *L. plantarum* X275 and *L. plantarum* X277 exhibited faster growth rates compared to other strains; additionally, the culture medium of *L. plantarum* X86 had the lowest pH value among all seven strains (Figures 3A,B).

**Table 2:** Information on *L. rhamnosus* LGG and the preliminary lactic acid bacteria strains.

Species	Strain	Morphology (on MRS agar)	Micromorphology
*Lacticaseibacillus rhamnosus*	LGG	Creamy white, round, smooth and of medium size	G+, rod-shaped
*Lactiplantibacillus plantarum*	X86	Creamy white, round, medium size	G+, short rod-shaped
*Lacticaseibacillus rhamnosus*	X130	Creamy white, round, medium size, irregular edges	G+, rod-shaped
*Lactobacillus paracasei*	X133	Creamy white, round, medium in size	G+, short rod-shaped
*Lactobacillus paracasei*	X135	Creamy white, round, medium in size	G+, short rod-shaped
*Lactobacillus paracasei*	X145	Creamy white, round, medium in size	G+, short rod-shaped
*Lactiplantibacillus plantarum*	X275	Creamy white, round, medium in size	G+, rod-shaped
*Lactiplantibacillus plantarum*	X277	Beige, round, medium size	G+, short rod-shaped

### 3.2 Screening of strains with probiotic effects

As shown in Figure 3C, the isolates showed varying tolerances to the artificial gastric and intestines juice. *L. plantarum* strains (X86, X145 and X275) demonstrated enhanced tolerance to artificial gastric juice at pH 3.0, with a survival rate of up to 70% after 3 h of treatment compared to other strains, while *L. plantarum* X86 and X133 exhibited the lowest survival rate (Figure 3D). The control strain LGG displayed the highest survival rate in both artificial gastric and intestinal fluid compared to the test strains. Among the *L. plantarum* strains (X275, X277 and X86), they showed superior bile salt tolerance with survival rates ranging from 63 and 80%, whereas *L. paracasei* X145 exhibited the poorest bile salt tolerance ability at only 20% (Figure 3E).

The growth of *S. aureus* strains SA2 and SA6 was significantly inhibited by the CFS, as demonstrated in Table 3. The inhibition rates for both strains ranged from 85.21–96.80% when the concentration of CFS was between 0.5–1.0. Furthermore, there was a noticeable decrease in antibacterial efficacy with lower concentrations of CFS. The CFS of lactic acid bacteria strains exhibited significant inhibitory effects on *S. aureus* biofilm, as indicated in Table 4. At concentrations ranging from 0.5 to 1.0, the inhibitory rates of two strains of *S. aureus* were between 88.39 and 101.23%. Additionally, at a concentration of 0.125, *Lactobacillus paracasei* X145 and *Lactobacillus rhamnosus* X130 still demonstrated inhibitory rates of 82.55 and 66.34%, respectively.

**Table 3:** Inhibition rate of Lactobacillus ssp. CFS against the growth of *S. aureus* strains (%).

*S. aureus* strains	CFS concentration	LGG	X86	X130	X133	X135	X145	X275	X277
SA2	1	95.00 ± 1.24^a^	95.05 ± 1.43^a^	95.27 ± 1.59^a^	95.10 ± 1.46^a^	95.25 ± 1.66^a^	95.49 ± 2.11^a^	95.05 ± 1.54^a^	95.12 ± 1.65^a^
	0.5	96.03 ± 1.02^a^	96.47 ± 1.00^a^	96.71 ± 1.21^a^	96.31 ± 0.79^a^	96.60 ± 1.12^a^	96.80 ± 1.33^a^	96.42 ± 1.32^a^	96.36 ± 1.27^a^
	0.25	2.32 ± 4.59^c^	65.21 ± 9.28^a^	67.99 ± 7.90^a^	63.32 ± 10.31^a^	51.91 ± 26.33^ab^	63.51 ± 13.59^a^	53.88 ± 15.66^ab^	37.14 ± 14.70^b^
	0.125	3.31 ± 4.41^c^	37.93 ± 7.73^a^	36.49 ± 13.62^ab^	22.11 ± 4.22^b^	31.53 ± 13.62^ab^	28.70 ± 12.42^ab^	11.60 ± 5.21^b^	25.72 ± 6.13^ab^
SA6	1	91.56 ± 4.50^a^	93.41 ± 1.19^a^	93.26 ± 0.99^a^	94.18 ± 2.25^a^	91.85 ± 4.69^a^	94.72 ± 2.70^a^	93.66 ± 1.57^a^	92.68 ± 1.35^a^
	0.5	90.32 ± 6.41^ab^	95.96 ± 1.00^a^	95.72 ± 0.65^a^	94.68 ± 1.29^a^	85.21 ± 13.23^b^	94.50 ± 1.55^a^	95.63 ± 1.36^a^	89.76 ± 9.41^ab^
	0.25	0.00 ± 0.00^c^	52.84 ± 11.31^a^	63.61 ± 9.91^a^	50.19 ± 9.88^a^	24.04 ± 21.24^b^	52.39 ± 13.75^a^	53.62 ± 9.47^a^	3.67 ± 5.80^c^
	0.125	2.48 ± 4.22^b^	29.28 ± 5.29^a^	34.44 ± 1.99^a^	27.07 ± 7.65^ab^	14.00 ± 15.49^b^	33.62 ± 2.92^a^	32.20 ± 13.66^a^	3.76 ± 5.52^b^

The data in the table were expressed as Mean ± SD, and group differences were assessed using Anova. Significant differences were denoted by different shoulder marks (*p* < 0.05), while the same shoulder marks indicated no statistically significant differences. CFS concentration 1 refers to the undiluted stock solution; 0.5–0.125 refer to different dilution concentrations of the CFS stock solution after 2, 4 and 8- times dilution, respectively.

**Table 4:** Inhibition rate of Lactobacillus ssp. CFS on biofilms from *S. aureus* (%).

*S. aureus* strains	CFS concentration	LGG	X86	X130	X133	X135	X145	X275	X277
SA2	1	91.97 ± 9.36^a^	95.40 ± 10.86^a^	96.97 ± 8.15^a^	96.14 ± 8.91^a^	89.78 ± 14.57^a^	99.81 ± 3.36^a^	89.13 ± 14.88^a^	98.30 ± 3.62^a^
	0.5	91.27 ± 8.82^a^	90.27 ± 10.79^a^	91.07 ± 6.27^a^	91.96 ± 11.72^a^	91.46 ± 8.29^a^	89.91 ± 9.54^a^	84.11 ± 21.96^a^	94.29 ± 2.85^a^
	0.25	76.02 ± 8.53^a^	74.44 ± 18.13^a^	84.59 ± 12.61^a^	79.58 ± 15.48^a^	0.00 ± 0.00^b^	79.64 ± 15.61^a^	79.07 ± 24.33^a^	83.50 ± 5.60^a^
	0.125	51.91 ± 8.90^b^	72.63 ± 8.32^ab^	77.50 ± 6.09^ab^	74.93 ± 15.24^ab^	0.55 ± 1.23^c^	82.55 ± 7.50^a^	73.49 ± 26.77^ab^	56.82 ± 27.55^ab^
SA6	1	91.65 ± 8.79^a^	91.42 ± 7.85^a^	92.89 ± 7.68^a^	99.87 ± 2.79^a^	92.72 ± 6.10^a^	101.23 ± 6.83^a^	93.64 ± 12.00^a^	99.12 ± 2.80^a^
	0.5	88.69 ± 9.51^a^	89.69 ± 8.53^a^	92.88 ± 7.24^a^	99.54 ± 2.21^a^	88.39 ± 13.98^a^	98.13 ± 8.07^a^	91.80 ± 9.56^a^	93.18 ± 11.15^a^
	0.25	49.21 ± 13.29^a^	0.00 ± 0.00^c^	60.54 ± 16.25^a^	48.16 ± 9.25^ab^	0.00 ± 0.00^c^	22.52 ± 6.77^b^	0.00 ± 0.00^c^	20.89 ± 24.75^b^
	0.125	0.68 ± 1.17^b^	0.00 ± 0.00^c^	66.34 ± 20.04^a^	53.63 ± 16.88^a^	0.32 ± 0.91^b^	3.22 ± 5.77^b^	0.00 ± 0.00^c^	2.27 ± 3.93^b^

The data in the table were expressed as Mean ± SD, and group differences were assessed using Anova. Significant differences were denoted by different shoulder marks (*p* < 0.05), while the same shoulder marks indicated no statistically significant differences. CFS concentration 1 refers to the undiluted stock solution; 0.5–0.125 refer to different dilution concentrations of the CFS stock solution after 2, 4 and 8- times dilution, respectively.

In addition, the adhesion of lactic acid bacteria to mammary epithelial cells was strain specific, and there was significant variation in adhesion ability even among different strains of the same species (Figure 3F). *L. plantarum* X86 and *L. paracasei* X133 exhibited the highest adhesion ability to MAC-T cells, with an adherent cell count of 10^8^ CFU/mL.

As shown in Supplementary Table S4, all isolates and *L. rhamnosus* LGG exhibited resistance to gentamicin, kanamycin, streptomycin and neomycin while demonstrating sensitivity to ampicillin, tetracycline, rifampicin and chloramphenicol (with the exception of *L. rhamnosus* LGG which was resistant to chloramphenicol). The susceptibility of lactic acid bacteria to lincomycin and erythromycin showed variability. Based on the above findings, it can be concluded that the lactic acid bacteria isolates had developed varying degrees of antibiotic resistance. Overall, *L. plantarum* X86 displayed the highest susceptibility among all tested antibiotics.

### 3.3 Screening score

The scores of isolates ranged from 63.57 to 76.20, with *L. plantarum* X86 achieving the highest score and *L. paracasei* X145 showing the lowest score (Supplementary Table S5).

### 3.4 The effect of *Lactobacillus plantarum* X86 against *Staphylococcus aureus*-induced mastitis rat model

#### 3.4.1 The mammary gland injury and pathological changes

**Figure 4:**
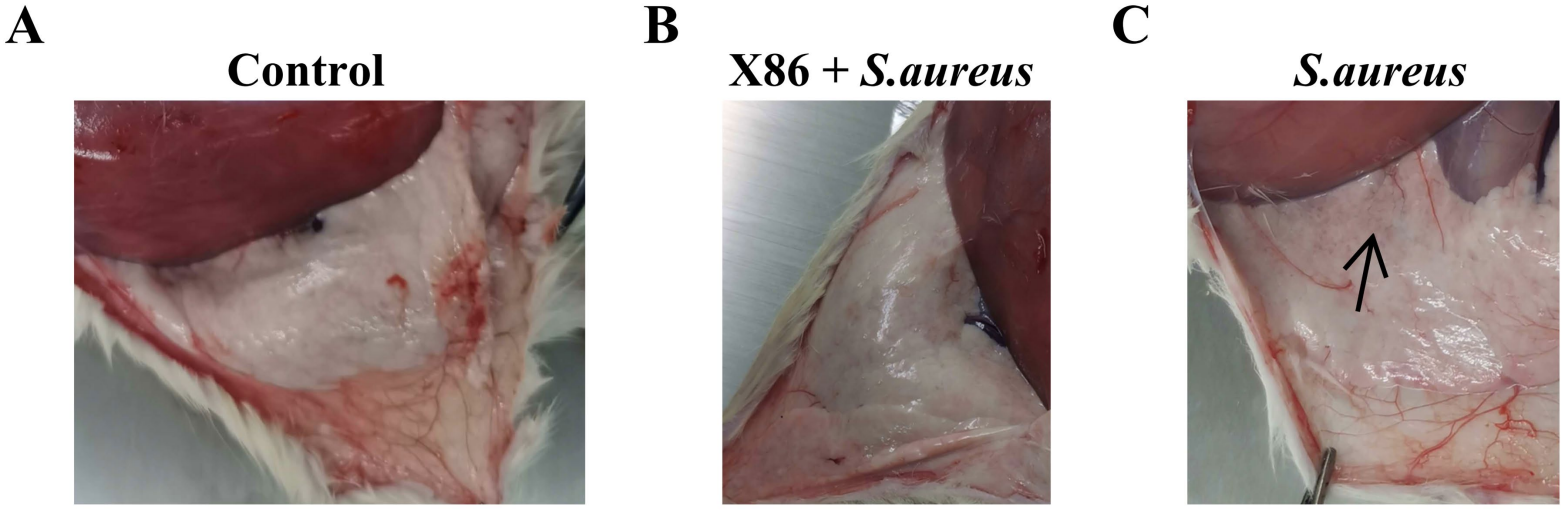
The morphological characteristics of mammary glands. **(A)** The Control group. **(B)** X86 + *S. aureus* group. **(C)** *S. aureus* group with hyperemia and presence of “particles” sensation (blank arrow).

**Figure 5:**
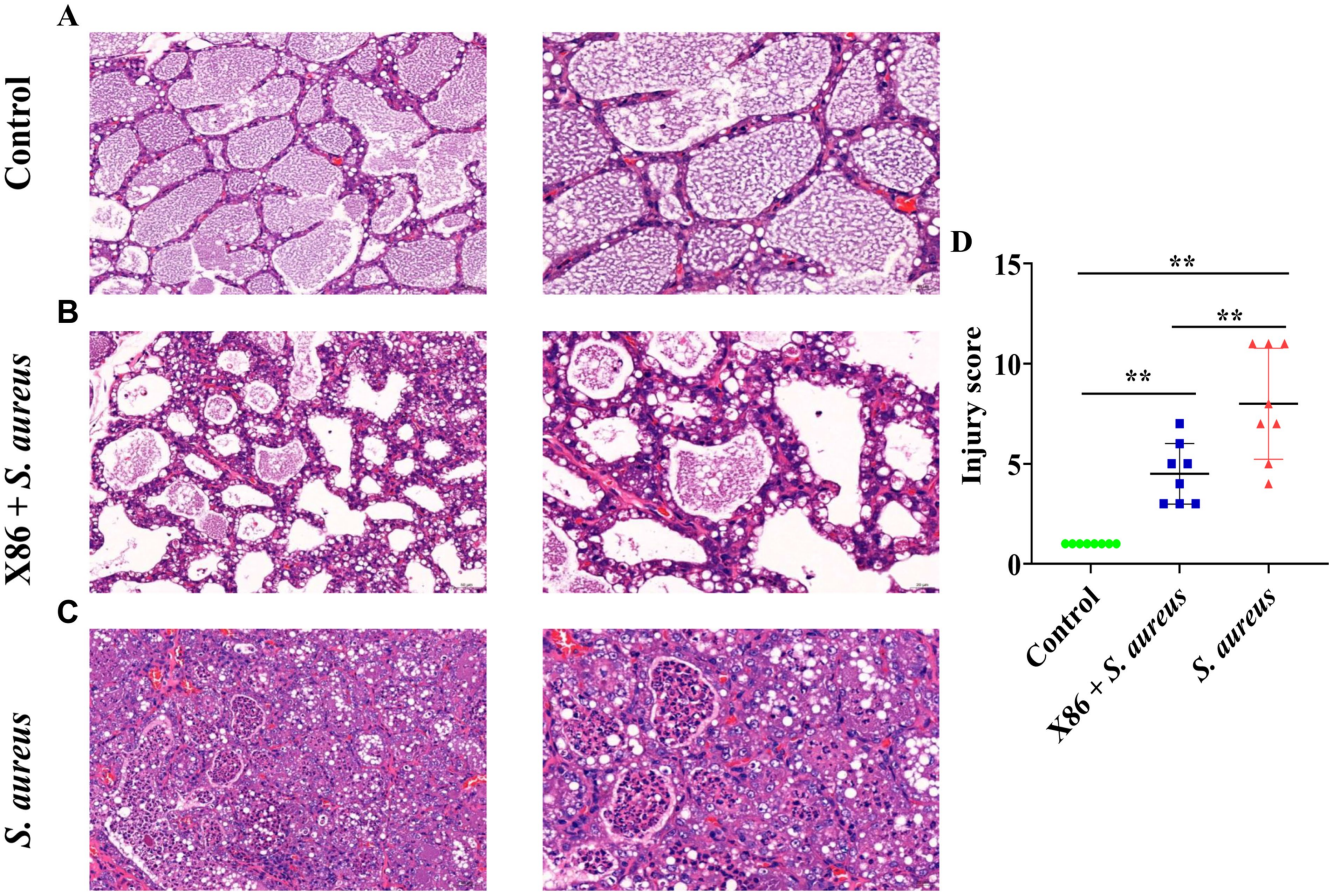
Hematoxylin and eosin (HE) staining of rat mammary gland. **(A)** Control group (left with 200×, right with 400×). **(B)** X86 + *S. aureus* group (left with 200×, right with 400×). **(C)** *S. aureus* group (left with 200×, right with 400×). **(D)** The comparison of the three groups. ** Indicates statistically significant difference (*p* < 0.01) (*n* = 8).

The mammary gland appearance was shown in Figure 4. The mammary gland areas of the rats in the X86 + *S. aureus* group and *S. aureus* group exhibited varying degree of hyperemia, with a consistency resembling “tofu residue.” In contrast, the mammary glands of rats in the Control group appeared smooth without evident congestion and displayed normal histology without inflammatory cell infiltration upon examination by H-E staining under a microscope. Conversely, within the *S. aureus* group, significant dilation of the mammary acini and ducts was observed, along with severe necrotic of epithelial cells and presence of numerous neutrophils and necrotic 1exfoliated epithelial cells within the glandular cavity. Furthermore, noticeable pathological damage was also evident in the mammary gland tissue of rats in the X86 + *S. aureus* group; however, there was a marked reduction in lesion severity compared to that seen in the *S. aureus* group alone (Figure 5).

#### 3.4.2 Cytokine and MPO concentrations in the mammary gland

**Figure 6:**
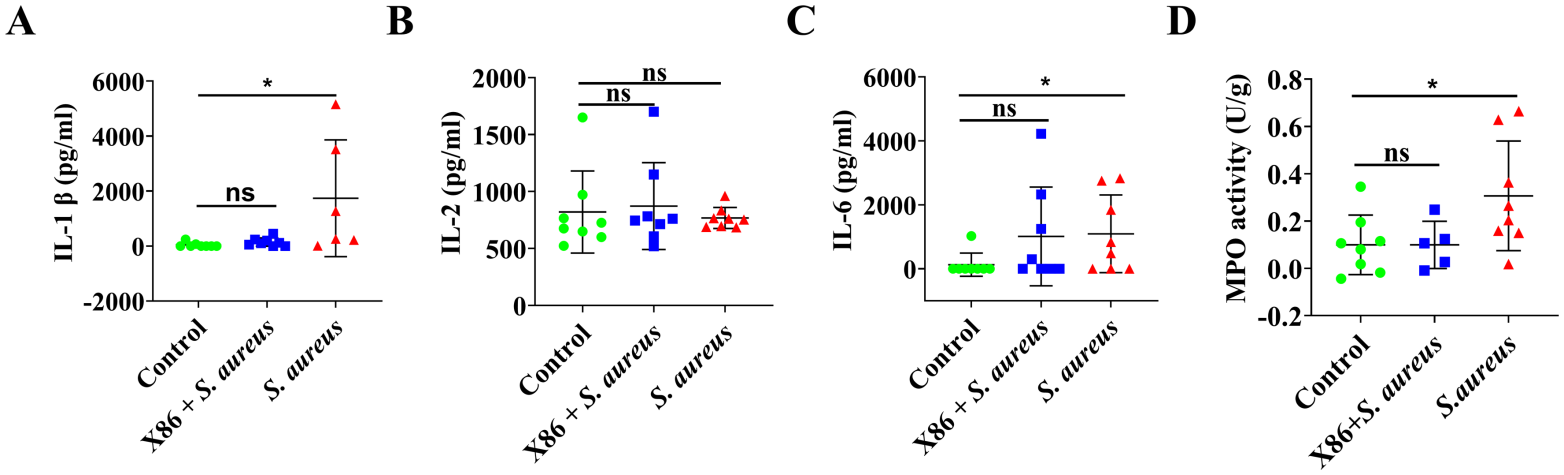
Elisa assay of proinflammatory cytokines and MPO activity test in rat mammary gland. **(A)** IL-1β. **(B)** IL-2. **(C)** IL-6. **(D)** MPO activity. The data were presented as Mean ± SD (*n* = 8), and inter-group differences were assessed using Anova. * Indicated a statistically significant difference (*p* < 0.05), while ns indicated no significant difference (*p* > 0.05).

ELISA kits were utilized for the detection of two pro-inflammatory cytokines and MPO activity in the mammary glands of rats within each experimental group. In comparison to the Control group, a significant increase (*p* < 0.05) was observed in the levels of IL-1β and IL-6 as well as MPO activity in the mammary glands of rats within the *S. aureus* group, while no significant difference was noted in the X86 + *S. aureus* group (*p* > 0.05) (Figure 6). These findings indicated that *L. plantarum* X86 had the potential to mitigate pro-inflammatory factors and MPO levels within the mammary gland, thereby exerting a protective effect against mammary inflammation at a protein level.

**Figure 7:**
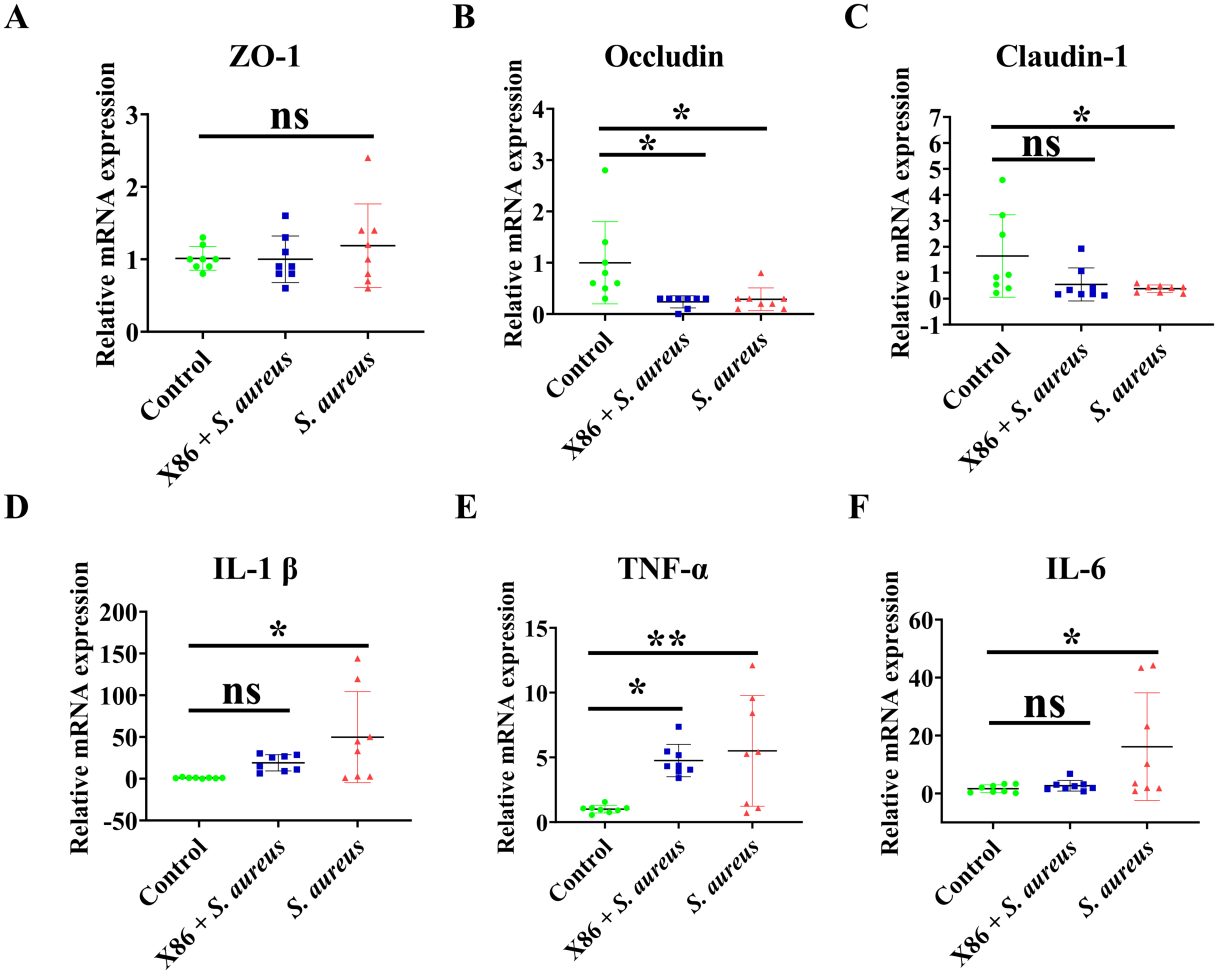
The mRNA expression of tight junction protein and proinflammatory cytokines in rat mammary gland. **(A)** ZO-1. **(B)** Occludin. **(C)** Claudin-1. **(D)** IL-1β. **(E)** TNF-*α*. **(F)** IL-6. The data were presented as Mean ± SD (*n* = 8), and inter-group differences were assessed using Anova. * Indicated a statistically significant difference (*p* < 0.05), ** indicated a statistically significant difference (*p* < 0.01), while ns indicated no significant difference (*p* > 0.05).

The mRNA expression levels of tight junction proteins ZO-1, Occludin, and Claudin, as well as proinflammatory cytokines IL-1β, TNF-*α*, and IL-6 in mammary gland tissues of rats in each group were detected using qRT-PCR. Figures 7A–C illustrates that there was no statistically significant difference in ZO-1 expression among the three groups (*p* > 0.05). However, Occludin expression was significantly decreased in both X86 + *S. aureus* group and the *S. aureus* group (*p* < 0.05), while Claudin-1 was significantly decreased only in the *S. aureus* group (*p* < 0.05).

The results of proinflammatory factor assays were showed in Figures 7D–F. As illustrated in the figures, there was no significant difference in the gene expression of IL-1β and IL-6 between the Control group and X86 + *S. aureus* group (*p* > 0.05). However, it is noteworthy that the gene expression of Il-1β and IL-6 in the *S. aureus* group exhibited a substantial increase (*p* < 0.05), with the highest observed fold changes being 144.1 and 44 times, respectively. Compared to the Control group, the X86 + *S. aureus* group exhibited significant increase in TNF-α levels (*p* < 0.05), while the *S. aureus* group showed an even more pronounced increase (*p* < 0.01).

#### 3.4.3 Changes of SCFAS content in rat feces

The fecal content of eight types of short-chain fatty acids (SCFAs) in the rats from each experimental group was analyzed using gas chromatography–mass spectrometry (GC–MS). As shown in Table 5, the X86 + *S. aureus* group exhibited the highest SCFAs content, surpassing that of the Control group and six types of SCFAs in the *S. aureus* group; however, this difference did not reach statistical significance (*p* > 0.05).

**Table 5:** The content of short-chain fatty acids (SCFAs) in rat feces.

Types of SCFAs		Group		Fold change (times)		
	Control	X86 + *S. aureus*	*S. aureus*	X86 + *S. aureus* vs. Control	X86 + *S. aureus* vs. *S. aureus*	Control vs. *S. aureus*
Acetic acid	0.62 ± 0.23^a^	0.73 ± 0.21^a^	0.67 ± 0.19^a^	1.2	1.1	0.9
Propanoic acid	0.60 ± 0.20^a^	0.73 ± 0.21^a^	0.50 ± 0.20^a^	1.2	1.5	1.2
Isobutyric acid	0.09 ± 0.04^a^	0.12 ± 0.03^a^	0.08 ± 0.04^a^	1.3	1.5	1.2
Butanoic acid	0.39 ± 0.20^a^	0.69 ± 0.41^a^	0.57 ± 0.47^a^	1.8	1.2	0.7
Isovaleric acid	0.06 ± 0.03^a^	0.08 ± 0.03^a^	0.05 ± 0.03^a^	1.4	1.6	1.2
Valeric acid	0.10 ± 0.05^a^	0.14 ± 0.05^a^	0.11 ± 0.07^a^	1.4	1.3	0.9
Isohexanoic acid	0.0022 ± 0.0006^a^	0.0025 ± 0.0010^a^	0.0025 ± 0.0015^a^	1.1	1.0	0.9
Hexanoic acid	0.03 ± 0.02^a^	0.07 ± 0.05^a^	0.08 ± 0.11^a^	2.5	0.9	0.3

The data in the table were expressed as Mean ± SD, and group differences were assessed using Anova. Significant differences were denoted by different shoulder marks (*p* < 0.05), while the same shoulder marks indicated no statistically significant differences.

## 4 Discussion

*S. aureus* is the most important pathogen responsible for dairy cow mastitis and has the ability to invade mammary epithelial cells and form bacterial biofilms, thereby impacting the efficacy of antibiotic treatment (40). Certain strains of lactic acid bacteria had been shown to possess antagonistic properties against pathogenic bacterial biofilm (41). In this investigation, all lactic acid bacteria strains exhibited inhibitory effects on the bacterial biofilms produced by *S. aureus* SA2 and SA6, albeit with slight variations in their inhibitory capabilities. This finding suggested that lactic acid bacteria in this study had the ability to antagonize *S. aureus* biofilm. Lactic acid bacteria establish colonization within the host organism through adherence to host cells and exert antagonistic effects against pathogens. In a previous study, different strains of lactic acid bacteria significantly reduced the adhesion rate of *S. aureus* to MAC-T cells (6). Nevertheless, a comparable observation was not identified in present study. One possible explanation might be that lactic acid bacteria, such as *L. plantarum* X86, adhere to cell surfaces via nonspecific mechanisms like surface electrostatic interactions rather than specific binding to cell receptors, thereby lacking competitive repulsion effects on the adhesion sites of *S. aureus* on cells.

In our study, perfusion of *S. aureus* in the mammary gland of rats resulted in pathological damage to the liver and intestine (colon), indicating that mastitis is not confined to local inflammation. However, administration of *L. plantarum* X86 via gavage significantly alleviated the pathological changes in these tissues, suggesting a preventive effect on mastitis in rats. It is worth noting that the pathological changes observed in tissues other than the mammary gland are unlikely to be directly caused by *S. aureus* itself, as the pathogen was not detected in the blood or feces of the rats. Furthermore, while *L. plantarum* was detectable in rat feces, it was not found in their mammary glands (data not shown). These results may suggest that the protective effect of *L. plantarum* X86 on the mammary gland did not stem from direct action by bacteria themselves but rather through stimulation of the host immune system responses and secretion of anti-inflammatory metabolites to prevent mastitis (42, 43).

In the early stages of mammary gland infection, epithelial cells’ pattern recognition receptors (PRR), such as Toll-like receptor 2 (TLR2), interact with microbe-associated molecular patterns (MAMP) to recognize pathogens, resulting in the secretion of cytokines like IL-1β and TNF-*α* and the recruitment of leukocytes (e.g., neutrophils) from the bloodstream to the mammary gland (10). Numerous studies had demonstrated that lactic acid bacteria can suppress NF-KB signaling activation (44, 45) and decrease proinflammatory factor expression in mouse mammary glands or BMECs (8, 10, 46). In the present study, *S. aureus* significantly elevated IL-1β expression (*p* < 0.05) and TNF-α expression of (*p* < 0.01) in the mammary gland, indicating the occurrence of mammary inflammation, while *L. plantarum* X86 inhibited the expression of these two cytokines to varying degrees. Furthermore, there were no changes observed in inflammatory factors with the gut (colon and jejunum). These results indicated that *S. aureus* mammary triggered inflammation in the mammary gland, but no significant change was observed in the intestinal tract. Analysis of serum inflammatory factors and MPO revealed no significant difference in the expression of pro-inflammatory factors IL-1β and TNF-α, as well as MPO levels in the bloodstream 24 h after infusion of *S. aureus* into mammary gland. The rapid onset of clinical symptoms following LPS infusion into cow’s mammary glands (47) was consistent with our results. The blood-milk barrier primarily consists of endothelial cells, connective tissue, basement membrane and epithelial cells. Occludin, ZO-1 and Claudin-1 are key tight junction proteins within the blood-milk barrier, with their expression levels serving as important indicators reflecting barrier integrity (11). In the current work, it was observed that the expression of Occludin gene in *S. aureus* group was significantly decreased compared to the control group (*p* < 0.05), while no change was noted in the expression of ZO-1 gene. Furthermore, *S. aureus* led to a reduction in the expression of Occludin, ZO-1 and Claudin-1 in the colon, with no significant changes observed in the jejunum. Interestingly, *L. plantarum* X86 demonstrated protective effects on breast epithelial cells against *S. aureus-*induced down-regulation of Claudin-1, as confirmed by immunohistochemistry (Supplementary Figure S1). These findings suggested that *S. aureus* had detrimental effect on both blood-milk barrier and intestinal barrier integrity. Previous studies had shown the ability of lactic acid bacteria to protect those barriers (48–50). It was worth noting that *L. plantarum* X86 exhibited strain-dependent weak protective effects on the blood-milk or intestinal barrier.

SCFAs, primarily composed of acetic acid, butyric acid and propionic acid, play a pivotal role in immune regulation and inflammatory states (38, 39). These SCFAs can enter host cells through transport or diffusion and interact with epithelial and immune cells by binding to G protein-coupled receptors such as GPR41, GPR43, and GPR109A (51). Furthermore, some SCFAs are capable of modulating the activation, recruitment, and differentiation of immune cells including neutrophils, DCs, macrophages and T lymphocytes leading to reduced expression of proinflammatory cytokines (9, 38). In a previous study, the authors observed a negative correlation between the levels of SCFAs in ileum mucosa and the proportion of associated opportunistic pathogens, suggesting their potential role in maintaining intestinal health in calves (52). Additionally, another study reported that the infusion of LPS into the mammary gland significantly decreased plasma level of SCFAs, especially propionate and butyrate (47). In the present investigation, it was observed that *S. aureus* perfusion led to a reduction in the levels of propionic acid, isobutyric acid and isovalaric acid in the rat intestine, indicating a close association between SCFAs content and mammary inflammation, which may impact the occurrence and outcome of mastitis. Previous study had demonstrated that SCFAs (butyric acid and propionic acid) possess inhibitory effects on pro-inflammatory cytokines production, activate the NF-κB signaling pathway, act as histone deacetylases in BMECs, and restore blood-milk barrier function. They played a protective role in LPS and *S. aureus*-induced mastitis models (53–56). In this study, it was found that *L. plantarum* X86 increased SCFA content in rats by approximately 1.8 times for butyrate, 1.4 times for valaric acid and 1.4 times for isovalaric acid. These findings suggested that *L. plantarum* X86 may modulate mammary gland inflammatory response of enhancing SCFAs, thereby potentially preventing *S. aureus*-induced mastitis in rats.

## 5 Conclusion

We characterized lactic acid bacteria strains derived from milk and identified a novel species of *L. plantarum* X86, which demonstrated resilience in gastrointestinal juice and bile salt, the ability to inhibit both planktonic and biofilm *S. aureus*, adherence rate to MAC-T cells, as well as the lowest antibiotic resistance. Additionally, *L. plantarum* X86 exhibited a preventive role in a rat mastitis model by reducing the inflammatory response and pathological damage while increasing the content of SCFAs.

## Data Availability

The original contributions presented in the study are included in the article/Supplementary material, further inquiries can be directed to the corresponding author/s.

## Footnotes

^1^ https://blast.ncbi.nlm.nih.gov/Blast.cgi

^2^ https://store.sangon.com/newPrimerDesign
