# Supplementary material for: The preventive effect of probiotic Lactobacillus plantarum X86 isolated from raw milk on Staphylococcus aureus-induced mastitis in rats
Source: Front Vet Sci. 2025 Mar 10;12:1476232. doi: 10.3389/fvets.2025.1476232 (PMC11931132; doi:10.3389/fvets.2025.1476232)
Supplement: Supplementary file 1 [file Table_1.docx]

**Table S1**

Antibiotic resistance cut-off value of lactic acid bacteria.

|  | *L. plantarum* | *L. rhamnosus* | *L. paracasei* |
| --- | --- | --- | --- |
| Ampicillin | 2 | 4 | 4 |
| Gentamicin | 16 | 16 | 32 |
| Kanamycin | 64 | 64 | 64 |
| Streptomycin | n.r. | 32 | 64 |
| Erythromycin | 1 | 1 | 1 |
| Clindamycin | 4 | 4 | 4 |
| Tetracycline | 32 | 8 | 4 |
| Chloramphenicol | 8 | 4 | 4 |
| Rifampicin | 4 | 2 | 2 |
| Neomycin | 32 | 8 | 4 |

The abbreviation n.r. indicates that testing is not required; The values in the table are determined in accordance with the guidelines provided by EFSA (EFSA Panel on Additives and Products or Substances used in Animal Feed (FEEDAP) et al., 2018) and supported by literature (ROZMAN et al., 2020; AMMOR et al., 2007; DANIELSEN & WIND, 2003).

**Table S2**

Screening score sheet for lactic acid bacteria.

| Level 1 | Assign points | Level 2 | Assign points | Grading rules |
| --- | --- | --- | --- | --- |
| Growth | 20 | Growth curve | 10 | At 9 h, OD600=1.38 gives 10 points. For each strain, the value decreased 0.1OD, minus 1 point; |
|  |  | pH curve | 10 | 10 points were assigned to pH=3.48 at 24 h, and 1 point was subtracted for every 0.1 increase in the value. |
| Basic prebiotics | 20 | Artificial gastric through | 5 | The survival rate of 20 was used as the score of this strain. |
|  |  | Artificial intestinal through | 5 | The survival rate of 20 was used as the score of this strain. |
|  |  | Bile salt tolerance | 10 | Survival rate of 10 was used as the score of this strain. |
| Against pathogens | 20 | Inhibition Planktonic bacteria | 10 | The concentration of CFS 0.5 was taken as the criterion, and the inhibition rate /10 was taken as the score of this strain (mean value of SA2 and SA6). |
|  |  | Inhibition biofilm | 10 | The concentration of CFS 0.5 was taken as the criterion, and the inhibition rate of 10 was taken as the score of this strain (mean value of SA2 and SA6). |
| Cell Adhersion | 20 | Cell Adhesion | 10 | Adhesion ratio of 10 was used as the score of this strain. |
|  |  | Adhesion inhibition | 10 | Adhesion inhibition rate of 10 was used as the score of this strain. |
| Antibiotic resistance | 20 |  | 20 | Each of the 10 antibiotics was given 10 points, a total of 100 points; If MIC exceeds X times of cut-off, X points will be deducted. If X≥10 times, 10 points will be deducted. Divide the sum of the scores by 5 for the final score. |
| Total | 100 |  | 100 |  |

**Table S3**

214 strains of lactic acid bacteria isolated from milk.

| Species | Number | Proportion（%） |
| --- | --- | --- |
| *Enterococcus hirae* | 64 | 29.91 |
| *Enterococcus faecium* | 56 | 26.17 |
| *Lactococcus garvieae* | 35 | 16.36 |
| *Lactococcus lactis* | 23 | 10.75 |
| *Weissella confusa* | 11 | 5.14 |
| *Lactiplantibacillus plantarum* | 9 | 4.21 |
| *Lacticaseibacillus paracasei* | 5 | 2.34 |
| *Enterococcus mundtii* | 4 | 1.87 |
| *Enterococcus sp* | 3 | 1.40 |
| *Weissella paramesenteroides* | 2 | 0.93 |
| *Weissella cibaria* | 1 | 0.47 |
| *Lacticaseibacillus rhamnosus* | 1 | 0.47 |
| Total | 214 | 100 |

**Table S4**

Antibiotic resistance of *Lactobacillus* ssp.

|  | GEN | AMP | KM | STR | CLI | ERY | TET | CM | NEO | RIF |
| --- | --- | --- | --- | --- | --- | --- | --- | --- | --- | --- |
| LGG | 64^R^ | 1^S^ | 1024^R^ | 128^R^ | <0.5^S^ | 0.5^S^ | 2^S^ | 8^R^ | 256^R^ | <0.5^S^ |
| X86 | 64^R^ | 0.125^S^ | 1024^R^ | n.r. | 2^S^ | 2^R^ | 16^S^ | 4^S^ | 128^R^ | 0.5^S^ |
| X130 | 64^R^ | 2^S^ | 1024^R^ | 128^R^ | <0.5^S^ | <0.5^S^ | 1^S^ | 4^S^ | 128^R^ | <0.5^S^ |
| X133 | 128^R^ | 1^S^ | 1024^R^ | 128^R^ | <0.5^S^ | <0.5^S^ | 2^S^ | 4^S^ | 256^R^ | <0.5^S^ |
| X135 | 64^R^ | 4^S^ | 1024^R^ | 256^R^ | <0.5^S^ | 0.5^S^ | 2^S^ | 4^S^ | 512^R^ | <0.5^S^ |
| X145 | 64^R^ | 4^S^ | 512^R^ | 256^R^ | <0.5^S^ | 8^R^ | 4^S^ | 2^S^ | 512^R^ | <0.5^S^ |
| X275 | 128^R^ | 1^S^ | >1024^R^ | n.r. | 8^R^ | 8^R^ | 32^S^ | 4^S^ | 512^R^ | <0.5^S^ |
| X277 | 64^R^ | 1^S^ | 1024^R^ | n.r. | 128^R^ | 512^R^ | 32^S^ | 4^S^ | 512^R^ | 0.5^S^ |

The abbreviation n.r. indicates that testing is not required; The values in the table represent the minimum inhibitory concentration, with the letter “R” representing resistance and “S” representing sensitivity. Sensitivity and resistance were determined in accordance with Table S1. GEN, Gentamicin; AMP, Ampicillin; KM, Kanamycin; STR, Streptomycin; CLI, Clindamycin; ERY, Erythromycin; TET, Tetracycline; CM, Chloramphenicol; NEO, Neomycin; RIF, Rifampicin.

**Table S5**

*Lactobacillus* ssp. score list.

| Level 1 | Level 2 | Points | LGG | X86 | X130 | X133 | X135 | X145 | X275 | X277 |
| --- | --- | --- | --- | --- | --- | --- | --- | --- | --- | --- |
| Growth and acid production | Growth | 10 | 9.56 | 9.70 | 8.52 | 6.49 | 5.19 | 4.86 | 9.94 | 10.00 |
|  | pH | 10 | 10.00 | 9.60 | 9.50 | 10.00 | 9.60 | 9.50 | 10.00 | 9.60 |
| Basic probiotics | Artificial gastric juice | 5 | 3.43 | 3.40 | 3.32 | 3.15 | 3.49 | 3.26 | 3.54 | 3.37 |
|  | Artificial intestinal juice | 5 | 3.55 | 3.05 | 3.48 | 3.10 | 3.29 | 3.63 | 3.61 | 3.63 |
|  | Bile salt | 10 | 4.81 | 6.43 | 3.01 | 4.30 | 3.14 | 2.06 | 8.34 | 7.67 |
| Antagonist is resistant to pathogenic bacteria | Planktonic bacteria | 10 | 9.32 | 9.62 | 9.62 | 9.55 | 9.09 | 9.57 | 9.60 | 9.31 |
|  | Biofilm | 10 | 9.00 | 9.00 | 9.20 | 9.20 | 8.99 | 9.40 | 8.85 | 9.37 |
| Cell adhesion and inhibition | Adhesion | 10 | 7.68 | 9.20 | 6.57 | 9.05 | 8.62 | 7.69 | 7.46 | 7.90 |
|  | Inhibition of adhesion | 10 | 0.00 | 0.00 | 0.00 | 0.00 | 0.00 | 0.00 | 0.00 | 0.00 |
| Antibiotic resistance |  | 20 | 14.00 | 16.20 | 14.40 | 14.80 | 14.80 | 13.60 | 12.00 | 11.20 |
| Score |  | 100 | 71.35 | 76.20 | 67.61 | 69.63 | 66.21 | 63.57 | 73.34 | 72.04 |
| Rank |  |  | 4 | 1 | 6 | 5 | 7 | 8 | 2 | 3 |
